# Some Prescriptions Discussed—II

**Published:** 1910-09-10

**Authors:** 


### THERAPEUTICS.

### SOME PRESCRIPTIONS DISCUSSED—II.

Two other good prescriptions for hypophosphites, each of which has been the result of much experimental work at the hands of pharmacists in Scotland, and each of which yields a sightly, stable, and, indeed, unexceptionable product, are the following:—

Syrupus Glycerophosphatum Simplex.

| | Parts. |
|---|---|
| Ferri glycerophosphatis ... ... ... | 0.25 |
| Magnesiæ glycerophosphatis ... ... | 0.50 |
| Sodii glycerophosphatis ... ... ... | 0.50 |
| Potassii glycerophosphatis ... ... | 0.50 |
| Acidi glycerophosphorici... ... ... | 0.50 |
| Caffeïn. alkaloid. ... ... ... ... | 0.25 |
| Sacchari ... ... ... ... ... | 70.00 |
| Spiritus chloroformi ... ... ... | 2.00 |
| Essentiæ vanillæ ... ... ... ... | 2.00 |
| Glycerini croci ... ... ... ... | 1.00 |
| Aquam cinnamomi ad ... ... ... | 100.00 |

and the stronger preparation of combined glycerophosphates and nux vomica:—

Syrupus Glycerophosphatum cum Nuce Vomicâ.

| | Parts. |
|---|---|
| Ferri glycerophosphatis ... ... ... | 0.50 |
| Magnesiæ glycerophosphatis ... ... | 1.00 |
| Sodii glycerophosphatis ... ... ... | 1.00 |
| Potassii glycerophosphatis ... ... | 1.00 |
| Acidi glycerophosphorici ... ... | 1.00 |
| Caffeïn. alkaloid. ... ... ... ... | 0.50 |
| Tincturæ nucis vomicæ ... ... ... | 8.00 |
| Sacchari ... ... ... ... ... | 70.00 |
| Persionis ... ... ... ... ... | 1.25 |
| Chloroformi ... ... ... ... | 0.50 |
| Spiritus rectificati ... ... ... | 2.50 |
| Aquam ad ... ... ... ... ... | 100.00 |

On the other hand, the practical dispenser finds objections to two other preparations of glycerophosphates advocated in the codex, namely, the following:—

Syrupus Glycerophosphatum Compositus, B.P.C.

| | Parts. |
|---|---|
| Calcium glycerophosphate ... ... | 2.00 |
| Potassium glycerophosphate ... ... | 1.00 |
| Sodium glycerophosphate ... ... | 1.00 |
| Magnesium glycerophosphate ... ... | 1.00 |
| Iron glycerophosphate (in fine powder) | 0.50 |
| Glacial acetic acid ... ... ... | 1.00 |
| Caffeïn. ... ... ... ... ... | 0.50 |
| Strychnine hydrochloride ... ... | 0.02 |
| Refined sugar ... ... ... ... | 70.00 |
| Cudbear ... ... ... ... ... | 1.25 |
| Chloroform ... ... ... ... | 0.25 |
| Alcohol ... ... ... ... ... | 0.50 |
| Distilled water, sufficient to produce ... | 100.00 |

It has been found very difficult to get the glycerophosphates and the caffein dissolved in the warm filtrate. When the sugar is added and heat applied, a solution is obtained; but when the product is left standing for a week, there is a considerable deposit. When the syrup is then filtered, a bright preparation results, but it is not up to the required standard as regards the percentage of calcium and of iron. Even if the sugar is dissolved without heat, the subsequent deposit still takes place; so that the prescription seems to be an imperfect one.

The same applies to another codex preparation of glycerophosphates, namely, the

Glycerinum Glycerophosphatum Compositum, B.P.C.

| | Parts. |
|---|---|
| Calcium glycerophosphate ... ... | 2.00 |
| Potassium glycerophosphate ... ... | 1.00 |
| Sodium glycerophosphate ... ... | 1.00 |
| Magnesium glycerophosphate ... ... | 1.00 |
| Iron glycerophosphate ... ... ... | 0.50 |
| Citric acid ... ... ... ... ... | 0.25 |
| Cudbear ... ... ... ... ... | 0.15 |
| Chloroform ... ... ... ... | 0.05 |
| Alcohol ... ... ... ... ... | 0.40 |
| Orange-flower water (undiluted) ... | 1.25 |
| Cherry laurel water ... ... ... | 2.00 |
| Glycerine ... ... ... ... ... | 50.00 |
| Distilled water, sufficient to produce ... | 100.00 |

The product of this prescription is satisfactory at first, but it tends to lose its bright colour on keeping, and it becomes muddy, a point which detracts much from its value.

The following elixirs all yield good preparations, and they are likely to be of practical service:—

Elixir Acetomorphinæ et Terpini, B.P.C.

| | Parts. |
|---|---|
| Acetomorphine hydrochloride ... ... | 0.10 |
| Terpin hydrate ... ... ... ... | 1.00 |
| Alcohol ... ... ... ... ... | 45.00 |
| Glycerin ... ... ... ... ... | 22.50 |
| Syrup of wild cherry, sufficient to produce ... ... ... ... ... | 100.00 |

A Scotch formulary of similar kind, containing however less acetomorphine and more terpin, is the following:—

Elixir Acetomorphinæ Compositum.

| | Parts. |
|---|---|
| Acetomorphinæ hydrochloridi ... ... | 0.083 |
| Terpinæ hydratis ... ... ... ... | 1.25 |
| Alcohol. (90 per cent.) ... ... ... | 25.00 |
| Extracti pruni virginiani liquidi ... | 16.60 |
| Essentiæ anisi (1 in 10) ... ... ... | 0.42 |
| Elixir glusidi ... ... ... ... | 0.42 |
| Glycerinum ad ... ... ... ... | 100.00 |

Each fluid drachm contains $\frac{1}{20}$ grain of acetomorphine hydrochloride and $\frac{2}{3}$ grain of terpin hydrate. The diminution in the former and the increase in the latter, as compared with the quantities in the codex elixir, are rendered possible by the substitution of glycerin for syrup. There is no crystallising out of the terpin from the preparation, and the taste is not so pungent as is that of some other combinations of a similar kind.

Another terpin elixir in local use is the following:—

Elixir Pumilio et Acetomorphinæ Compositum.

| | Parts. |
|---|---|
| Olei pini pumilio ... ... ... ... | 1.60 |
| Terpinæ hydratis ... ... ... ... | 1.60 |
| Acetomorphinæ hydrochloridi ... ... | 0.083 |
| Alcohol ... ... ... ... ... ... | 30.00 |
| Glycerini croci ... ... ... ... | 1.25 |
| Glycerinum ad ... ... ... ... | 100.00 |

One drachm contains 1 minim of the pumilio pine oil, 1 grain of terpin, and $\frac{1}{20}$ grain of acetomorphine hydrochloride. It contains less pine oil and more terpin and acetomorphine than does the codex elixir. Replacement of the syrup by the glycerin ensures a clear and stable preparation.

Elixir Quininæ Ammoniatum, B.P.C.

| | Parts. |
|---|---|
| Quinine sulphate ... ... ... ... | 1.00 |
| Ammonium carbonate ... ... ... | 3.00 |
| Alcohol ... ... ... ... ... ... | 25.00 |
| Solution of carmine ... ... ... | 0.25 |
| Elixir of orange ... ... ... ... | 50.00 |
| Distilled water, sufficient to produce ... | 100.00 |

If the quinine sulphate is diffused in the alcohol, the ammonium carbonate dissolved in water, and the two mixed, solution takes place almost immediately.

There are certain difficulties in connection with the two following codex preparations:—

Mistura Bismuthi Composita cum Pepsino, B.P.C.

| | Parts |
|---|---|
| Bismuth citrate ... ... ... .. | 9.00 |
| Solution of ammonia ... ... ... | a sufficiency |
| Pepsin ... ... ... ... ... | 2.00 |
| Chloroform ... ... ... ... | 1.00 |
| Tincture of nux vomica ... ... ... | 12.50 |
| Diluted hydrocyanic acid ... ... | 3.25 |
| Solution of carmine ... ... ... | 0.75 |
| Distilled water, sufficient to produce ... | 100.00 |

This mixture contains, in each fluid drachm, about 5 grains of bismuth citrate, 1 grain of pepsin, 7½ minims of tincture of nux vomica, and 2 minims of dilute hydrocyanic acid; but after it has been made up a week or two, precipitation sets in, and the particles that are deposited carry with them a considerable portion of the colouring matter. Even after filtration the preparation is not bright and clear like a well-known proprietary article; and filtration must be objected to, because it alters the proportions of the ingredients. It has been suggested that the alcohol is to blame, and that it might be better to leave out the tincture of nux vomica and the chloroform, and use equivalent amounts of the liquid extract of nux vomica and of chloroform water.

Pilulæ Podophylli Compositæ, B.P.C.

| | Parts. |
|---|---|
| Podophyllum resin ... ... ... ... | 15.00 |
| Mercurous chloride ... ... ... | 60.00 |
| Alcoholic extract of belladonna ... | 10.00 |
| Milk sugar ... ... a sufficiency to | 100.00 |
| Syrup of glucose ... a sufficiency to | 100.00 |

Each pill contains about ¼ grain of podophyllum resin, 1 grain of calomel, and ⅛ grain of alcoholic extract of belladonna, but the pills are very difficult to make according to the above formulary, because the pill-mass is not sufficiently adhesive; it is suggested that it would be an improvement to add 5 parts of tragacanth to the prescription.

